# Typhoid Fever

**Published:** 1880-10

**Authors:** Austin Flint

**Affiliations:** Professor of the Principles and Practice of Medicine in the Bellevue Hospital Medical College, New York


### Selections.

### TYPHOID FEVER.

BY AUSTIN FLINT, M.D.,
Professor of the Principles and Practice of Medicine in the Bellevue Hospital Medical College, New York.

GENTLEMEN :—To-day I will present to you a case which I know will interest you, and I will state at the outset that it is one of typhoid fever. It may be a question whether it is not one of typho-malarial fever. Our suspicion of the existence of this is obtained principally from the previous history of the case. At the present time we are seeing cases of typhoid and malarial fevers combined. These two fevers may be combined in a given case in varying proportions. In the present case an inference of the coexistence of these two diseases is based rather upon the history given by the friends of the patient, and also because she has been treated for some time pretty largely with quinia.

I will first of all ask your attention to the answer of this question : "On what grounds do we conclude that this patient has typhoid fever?" The patient is a young subject, twenty-seven years of age, Annie O'D———, and was admitted to Bellevue Hospital, September 7, 1880. I will confine my remarks for the present to the above question. This patient was confined with a still-birth ten weeks before her admission. She recovered from that, and, as far as we can learn, her present illness began about three weeks before she entered the hospital ; she had been confined to her bed for about two weeks previous to her admission. Now this slow development of the disease is one point in favor of typhoid fever. Typhoid developes in this slow way—so slowly that the patients cannot tell exactly when they began to be sick. Usually it is from four to ten days from the beginning of this disease before the patient is obliged to take to bed. I am in the habit of calling the time when the patient takes to bed as the time when the disease begins, as the prodromic period is likely to vary considerably. This slow development of the disease is characteristic of typhoid fever; malarial fever and other fevers do not have this long prodromic period.

Since her admission she has at times manifested an inclination to cough, and upon examination, a little bronchitis was found to exist; this also goes with typhoid fever. She complained of pain in the abdomen, and at that time her bowels appeared to be constipated. It was ascertained that she had been bleeding at the nose—epistaxis—a trifling event when taken alone, but taken in connection with the other symptoms which are present, it is of considerable importance. Epistaxis not infrequently occurs in the early part of typhoid fever. At the time of her entrance to the hospital her temperature was found to be 103°, and on the following morning it was 104°. In the evening the temperature was the same, 104°, pulse, 100. On the next day in the morning her temperature was 103°. No medicinal treatment was entered upon. She was deaf and the deafness could not be explained as being due to the administration of quinine ; so we have another symptom of typhoid fever. I have known almost complete deafness to occur in typhoid fever. On the day she entered the hospital she had had two movements of the bowels. They were loose and of a yellowish color. Of course this alone would be of little or no value, but taken in consideration with other symptoms which were present in her case, it is of diagnostic value. On the 9th of September her temperature was 103° in the morning. She was now placed upon half an ounce of whiskey every four hours, together with milk as a diet. She has also had aromatic spirits of ammonia, which, though not a very potent remedy, is still a remedy of some value. The urine was examined, but nothing of importance was found. Upon examination of the abdomen externally it was found somewhat tympanitic, owing to the accummulation of gas in the intestines, and there was distinct tenderness in the iliac region upon the right side ; this was diagnostic. We looked of course for the eruption, but the eruption was not present then, nor has it been present since. The absence of the eruption, however, is not very infrequent in cases of typhoid fever. The patient has been under observation since September 7th, during which time there has been a typhoid condition of the mind—a passive delirium, attended with some incoherency and an indifference concerning persons and things. She has had all along the same appearance which you see now in her physiogomy. This appearance is not as marked now as it has been in the ward, owing to the excitement of the mind of a woman upon being brought into the presence of so many gentlemen; but, after all, she manifests very little disturbance.

These, then, are the points by which we may make the diagnosis of typhoid fever in the case before you; the slowness of its development, the existence and the continuance of the fever, with not much variation between the morning and evening temperatures, not so much oscillation as is found in malarial fever, the occurrence of epistaxis early in the disease, a pulse with little fluctuation, the passive delirium, the dullness of the intellect, the existence of tympanitis, the loose passages from the bowels and the existence of bronchitis.

The husband of this patient stated, that in the house whence she came, there exists an unpleasant condition of the sewers. We have also learned, that, in the same house, another individual has been attacked much in the same way and with apparently the same disease as the patient before you. So here are facts which seem to show the source of the disease to be defective sewerage. So much for the diagnosis and causation.

This patient has now been in the hospital ten days, and she was ill two weeks before she entered the hospital; that is, was confined to her bed, so by this estimate she is now in the twenty-fourth day of the disease. With regard to the duration of typhoid fever, I would say, that it is a self-limited fever. It has a law of duration, yet the duration varies within tolorably wide limits. If the fever be of short duration, it will last not much less than fourteen days. The longest time I have ever known it to continue is ninety days, and this was in a case in the hospital, years ago. In the case before us it is evident we have not reached the end of the fever, and it is now about the twenty-fourth day.

I will now pass to another very interesting aspect of the case, and I have so much matter that I am a little embarrassed as to how to present it to you. There has been made a record of the temperature, respiration and pulse of this patient for nearly every hour since she entered. It would be tedious to read all this, and I will, therefore, endeavor to abstract those facts which are of especial interest in reference to treatment. The interest of these facts has relation especially to the antipyretic treatment of typhoid fever and the importance of the application of cold to the surface of the body as an antipyretic measure. The antipyretic treatment of fevers has apparently wrought an important change in practical medicine. Fevers may be diminished in several ways. A large dose of quinia will do it in some instances, but it cannot be relied upon. Quinia has not been employed in this instance. More reliable is cold applied in the form of the cold bath, the cold sheet, or the wet pack. The cold bath was first used, but as it is a troublesome method and frequently produces a nervous shock to the patient, it has been found, on this account, desirable to use a substitute for it. I believe we we can procure the same results from the cold sheet, and by that I mean the wrapping of the body in a wet cloth and sprinkling with cold water. The wet pack, although an antipyretic measure, does not accomplish much through the agency of cold, as the action of the cold is continued only for a few minutes, the body being wrapped in a wet sheet and a thick, dry covering placed over all; the good effects obtained from the pack is through other means than by cold. There is still another way of applying cold to the surface of the body, which is of all means the easiest, viz., spronging the entire surface with cold water. If the sponging be practised thoroughly, it is quite effective. Where the circumstances will admit, you can denude the whole body and sponge it thoroughly, or where this is not admissible, you may unrobe a portion of the body, and after this another part, and so on until the entire surface has been sponged. The object in general terms is this: Where there exists a hyperpyrexia, that is, a temperature of 103° or over, some method of applying cold is to be employed. The patient should be carefully watched and perhaps given a little alcoholic stimulus. After the cold has been applied for a time, the patient should be placed in bed and the temperature taken, so as to determine what effect, if any, the bath has had. The temperature of the body often falls for some time after the application of cold has been suspended. It is desirable to bring the temperature down to 101° or lower. When the temperature again rises, application of the cold is again desirable, under the theoretical view, that much of the danger is in some way dependent upon the degree of the fever, and, although we do not abort the fever, we abate it. I fully believe in this, and I believe it to be a very important progress in the treatment of essential fevers and certain acute inflammations. Now let us see, this is a modern treatment, and yet it is the revival of a very old treatment. Nearly a century ago, an English physician, Currie, recommended this treatment, and if you have the opportunity of reading his work I would advise you to do so, for it is a work which bears the evidence of great honesty. He claims that in a large majority of cases he could arrest continued fevers. He employed cold water in the form of the douche, or cold affusion. I suppose, perhaps, the apparent severity of this measure led to its becoming obsolete; but Currie anticipated what we recommended to-day. It is further interesting to note, that he devised an axillary thermometer, which was bent so as to be placed under the arm; in fact, he anticipated the modern use of the thermometer, as well as the use of cold in fevers.

Now let us see what we can find, in the history of this case, that is instructive in the employment of cold in the treatment of this disease. I would state that half an ounce of whiskey every four hours, and afterward the same quantity every three hours, and now every two hours, has been given; and that, in connection with baths and a milk diet, has constituted the treatment. She has also taken aromatic spirits of ammonia, which, however, is not a very potent remedy. I will try not to be tedious, but limit myself to points which are instructive.

Well, she had a sponge-bath September 10th. This was employed about twelve o'clock (noon), and at half-past twelve her temperature was 103¼°. An hour before the bath, at eleven o'clock, it was 103½°, a reduction of only one-fourth of a degree. At forty-five minutes past one, another sponge-bath was given, and at two o'clock her temperature was 103¾°; no result thus far. At 2.45 P.M. the temperature was 104°, and at three o'clock the patient was placed in a cold pack for an hour. At four o'clock in the afternoon, her temperature was 102½°; the reduction evidently being from the effect of the cold, because the temperature would naturally increase rather than decrease in the afternoon.

At ten o'clock on the morning of the 11th her temperature was 103¼°; a sponge-bath was given, and at 10 30, her temperature was 102°—a sudden reduction of a degree and a quarter due to the application of the sponge-bath. At one o'clock, her temperature was 102¼°, and at four o'clock her temperature was 104°. A sponge-bath was given and at 4.45 her temperature was 102°, a reduction of two degrees. At seven o'clock her temperature was 104° Ten minutes after seven a sponge-bath was given, and at 7.40 P.M., her temperature was 103°. At ten o'clock in the evening, her temperature was 104°; she was given a sponge-bath but its effect was not recorded.

September 12th, at 1 A.M., her temperature was 104½. She was given a sponge-bath, and at 2 30 A.M., her temperature was 104°; practically no effect. These figures are instructive from their negative, as well as from their positive evidence. How long the bath was continued is not stated. At three o'clock she was placed in a cold pack for an hour, and at four o'clock her temperature was 102½°. At seven o'clock her temperature was 103¼°, and then a sponge-bath was resorted to, and at eight o'clock her temperature was 102½°; it rose again however, and at ten o'olock was 103°. A sponge-bath was given and the temperature fell to 102¼°.

Now I come to the record of September 13th. At ten o'clock in the morning her temperature was 103¼°. At 10.50 A.M., it had fallen one-fourth of a degree. At one o'clock, her temperature was 104°; a sponge-bath reduced it to 103½°. At 3.45 P.M., her temperature was 105⅛°. At 5.30 P M., the patient was placed in a cold pack, and at 6.30 P.M., her temperature was 103¾°. At nine o'clock, her temperature was 104⅛°. A cold pack was given, and at ten o'clock it was 102½°. At twelve o'clock the temperature had risen and the cold pack was resorted to, but with what result is not recorded.

September 14th, 2 A.M, temperature 104°, two hours after the cold pack on the previous day. Cold pack was administered, and at six o'clock her temperature was 103°, illustrating that the decline of temperature after the application of cold does not always take place immediately.

September 15th, at midnight, her temperature was 104°. A cold pack was given about one o'clock in the morning, which reduced the temperature to 103¼°. At 4 P.M., September 15th, her temperature was 103¼°. A cold pack was administered, and at seven o'clock it was 103⅛°; only a slight reduction.

September 16th, at one o'clock in the morning, her temperature was 103¼.° A bath was given, and by 6 A.M., the temperature had fallen to 102½°. On the 16th, finding her respiration somewhat quick (forty-two to the minute, while the pulse was only a hundred,) I examined this patient, believing this disproportion between the heart-beats and the respiratory movements to indicate some pulmonary difficulty. I suspected pneumonia, which, however, was somewhat inconsistent with the temperature; but upon examination of the chest, I found evidences of bilateral œdema in the posterior aspect of the lungs, which was due to the gravitation of the blood into that part from the continued recumbency upon the back. The evidences of this were fine bubbling rales. This condition pointed to rather an important point in the treatment of typhoid fever, namely, not to permit the patient to rest upon the back all the time.

Now we come to the 17th of September and that is today. Her temperatnre at two this morning was 102°; at five o'clock, 102°; at eight o'clock 102½°; and at 11.45 A.M., 103°. Thus far to-day there has been no application of cold for the reason that there has been no hyperpyrexia

The temperature has been under 103°, with the exception of 11.45 A.M., when it was at that point.

Now, gentlemen, you will see by these details that reduction of temperature does not always follow the application of the sponge-bath, but that very generally the wet pack has reduced the temperature one or two degrees. Perhaps this has not been accomplished quite as fully as we might desire, but the febrile condition has been kept down within the limits of hyperpyrexia, namely, at a temperature of about 103°. The treatment, aside from this, please bear in mind, has consisted of alcohol as a sustaining agent, with milk for diet, and except in those cases where there is a repugnance to milk, this, as an article of diet, is to be preferred.

